# Biomimetics provides lessons from nature for contemporary ways to improve human health

**DOI:** 10.1017/cts.2021.790

**Published:** 2021-05-17

**Authors:** Peter Stenvinkel, Carla M. Avesani, Line J. Gordon, Martin Schalling, Paul G. Shiels

**Affiliations:** 1 Division of Renal Medicine, Department of Clinical Science, Technology and Intervention, Karolinska Institutet, Stockholm, Sweden; 2 Stockholm Resilience Centre Stockholm University, Stockholm, Sweden; 3 Department of Molecular Medicine and Surgery, Karolinska Institutet, Stockholm, Sweden; 4 Institute of Cancer Sciences, Wolfson Wohl Translational Research Centre, University of Glasgow, Bearsden, Glasgow, UK

**Keywords:** Biomimetics, planetary health, Nrf2, oxidative stress, biodiversity, food systems, COVID-19

## Abstract

Homo sapiens is currently living in serious disharmony with the rest of the natural world. For our species to survive, and for our well-being, we must gather knowledge from multiple perspectives and actively engage in studies of planetary health. The enormous diversity of species, one of the most striking aspects of life on our planet, provides a source of solutions that have been developed through evolution by natural selection by animals living in extreme environments. The food system is central to finding solutions; our current global eating patterns have a negative impact on human health, driven climate change and loss of biodiversity. We propose that the use of solutions derived from nature, an approach termed biomimetics, could mitigate the effects of a changing climate on planetary health as well as human health. For example, activation of the transcription factor Nrf2 may play a role in protecting animals living in extreme environments, or animals exposed to heat stress, pollution and pesticides. In order to meet these challenges, we call for the creation of novel interdisciplinary planetary health research teams.

## Introduction

**Fig. 1.**
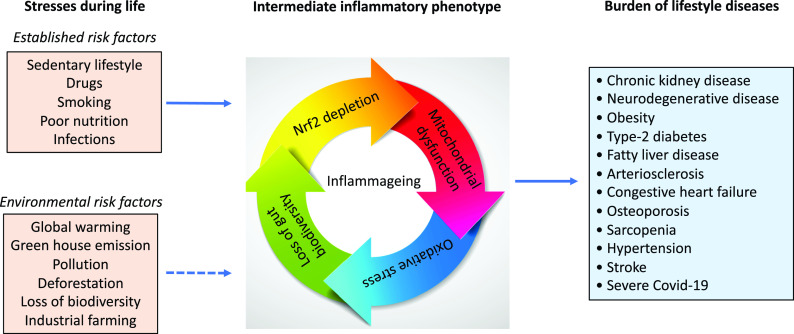
Numerous life stressors activate the inflammatory intermediate phenotype associated with an imbalanced microbiota, oxidative stress and mitochondrial dysfunction, which drive the risk of burden of lifestyle diseases that accumulate with age. Besides established risk factors, emerging evidence suggests factors that are related to our external environment, such as global warming, deforestation, pollution also increase the risk of burden of lifestyle diseases and contribute to inflammageing.

Global health is rapidly being challenged by an aging population and epidemics of burden of lifestyle diseases that accumulate with age such as type-2 diabetes, obesity, non-alcoholic fatty liver, arteriosclerosis, depression, neurodegenerative diseases, hypertension, congestive heart failure (CHF), chronic kidney disease (CKD), cancer, chronic pulmonary disease, stroke and osteoporosis [1]. This rapidly growing group of chronic diseases is characterised by a low-grade chronic inflammation [2], termed inflammageing [3], mitochondrial dysfunction [4] and oxidative stress with increased generation of reactive oxygen species (ROS) [5] that accompanies the aging process (Fig. 1). These features are, in part, reflected by the repressed activity of a master regulator of hundreds of cytoprotective genes. This is the transcription factor “*nuclear factor erythroid 2-related factor 2*” (Nrf2), and its inhibitor kelch-like ECH-associated protein 1 (Keap1), which protects against inflammation and oxidative stress when upregulated [6]. Age-related chronic disease is also commonly characterised by a loss of gut microbiota biodiversity [7]. The emerging epidemic of burden of lifestyle diseases in modern society is partly due to genetic, epigenetic and functional adaptations that have taken place during evolution, as a result of changes in human development in response to changes in climate, access to food and pandemics. Thus, to handle the epidemics of burden of lifestyle diseases, we need to understand the history of our planet and the changes that occurred during evolution [8]. Notably, we need a better understanding of how our health is influenced by our exposome across the human life course. The exposome comprises the totality of human environmental (both biotic and abiotic) exposures from conception to death [9]. The importance of the human exposome is emphasised by the finding that three simple exposome factors, namely air pollution, tobacco smoke and diet, account for ∼50% of mortality globally [10]. How such factors interplay, either cumulatively, independently or synergistically, with human genomes and epigenomes is poorly understood, especially in the context of antagonistic pleiotropy and psychosocial biology [11]. Thus, while the ongoing environmental crisis will undoubtedly have a significant impact on human health spans, identifying and mitigating the adverse effects of exposome dynamics at different life stages may take generations.

**Fig. 2.**
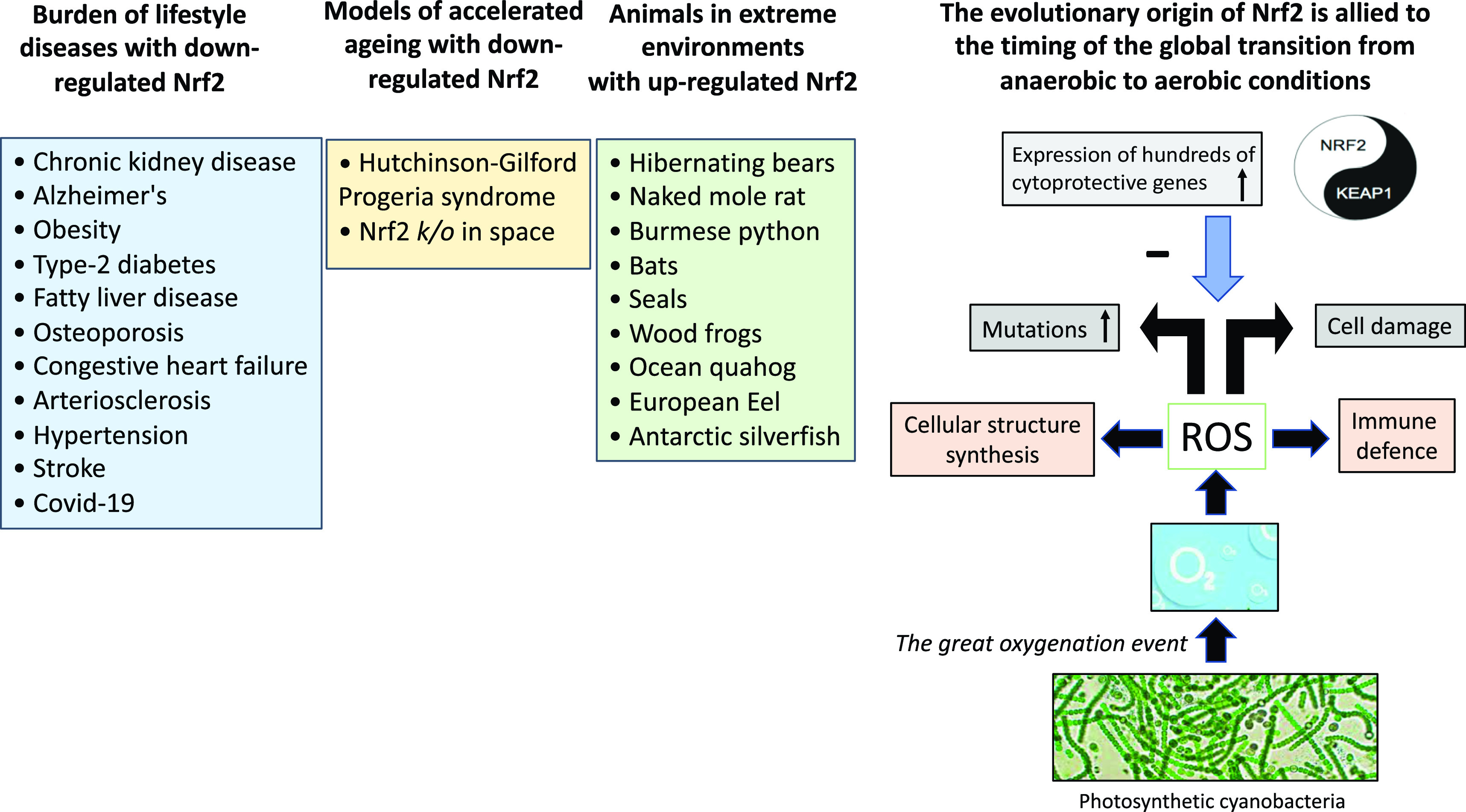
The “*Great Oxygenation Event*” set the stage for the evolutionary transition to the aerobe-dominated biota that still continues and the evolutionary origin of Nrf2. When living organisms were exposed to oxygen (released from photosynthetic cyanobacteria), a need emerged for protection against cell-damaging reactive oxygen species. The master switch and transcription factor Nrf2 emerged for cytoprotective activation with an inhibitor Keap1 that fine-tuned the activity of Nrf2. Based on studies in animal species, it can be hypothesised that superior anti-oxidant defence mechanisms with enhanced Nrf2 expression are protective and facilitate survival in extreme environments. Experiences from rare progeric diseases, such as Hutchinson-Gilford Progeria syndrome and Nrf2 *k/o* mice stressed by space travel, imply a protective role of Nrf2 in aging. Given the association between depressed expression of Nrf2 and chronic burden of lifestyle diseases associated with inflammation and oxidative stress that accumulate with aging, nutrients targeting Nrf2, using “Food as Medicine“ [127], may have a positive effect on a cluster of burden of lifestyle diseases and planetary health. ROS, reactive oxygen species.

**Fig. 3.**
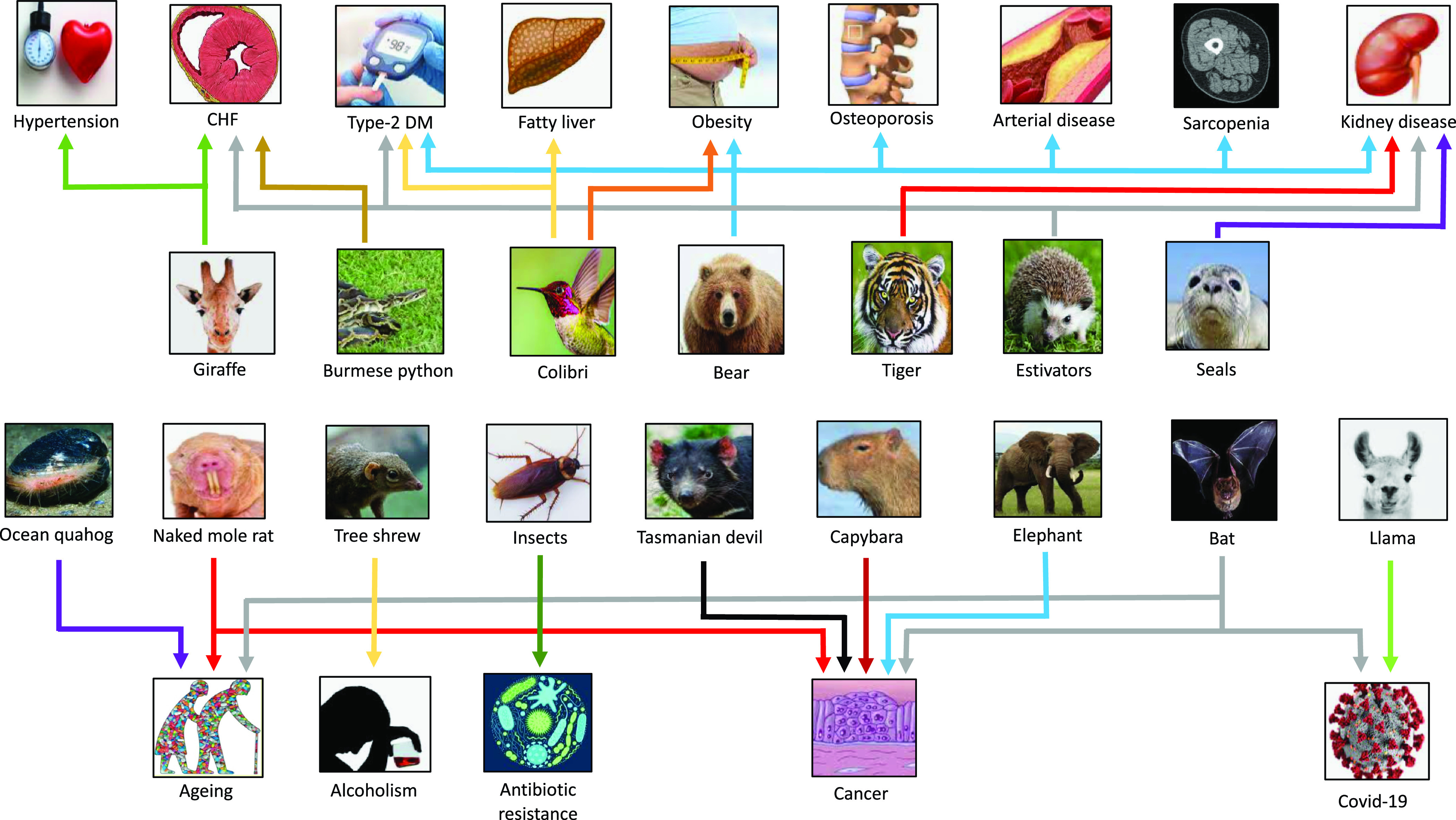
Selected examples of species that have provided clues for novel targets for “burden of life style” diseases, aging, alcoholism, COVID-19 and antibiotic resistance. CHF, congestive heart failure; DM, diabetes mellitus.

About 3.5 billion years ago (BYA), life on the planet was created from the sea [12]. A vital challenge, key to the success of the history of aerobic land life on our planet, is how cellular processes co-adapted to overcome the metabolic toxicity that results from the presence of ROS. The “*Great Oxygenation Event”* about 2.4 BYA, is recognised as the most geologically critical environmental change since it submersed the planet in an oxidising atmosphere, which set the stage for the evolutionary transition to the aerobe-dominated biota that still exists [13]. As a waste product of the planets most successful group of microorganisms (photosynthetic cyanobacteria) excess oxygen was released that induced the production of ROS, that benefit the immune defence and cellular structure synthesis. However, as a double-edged sword, elevated levels of ROS may also lead to cell damage and it has been proposed that ROS acted as a primary driver of evolution since it causes mutations in the genome and induces irreversible oxidative modification of lipids, glycans and proteins [5]. Orthologues of Nrf2 first appeared in fungi; thus, it is believed that Nrf2 evolved as a protection when organisms were exposed to oxygen and ROS [14]. Understanding the evolution of Nrf2 as an effective antioxidant response to various stressors in plants, animals and humans on the planet, has major implications for the ongoing epidemic of burden of lifestyle diseases associated with inflammageing and reduced mitochondrial biogenesis (Fig. 2). In this review, we discuss examples of animals that may provide novel solutions for prevention and/or treatment of burden of lifestyle diseases (Fig. 3).

## Transformation of Planets Natural Systems: We Face a Different Disease Panorama

The accelerating disturbances in the planet’s external environment will perturb interactions between a disturbed internal and external milieu, effectively increasing the chronic inflammatory burden of lifestyle diseases (i.e., a disturbed “internal environment”). It is critical that we understand these relations in order to reduce the burden of illness. Studying these complex interactions requires a syndemic approach that integrates studies of human health and animal health, with changes in our external environment; that is, “Planetary health” [15] defined as “*the health of human civilisation and the state of the natural systems on which it depends*.*”* It is likely that a disturbed planetary health will have a more severe impact on susceptible individuals such as children, the elderly and patients with chronic debilitating disorders. Thus, we need to understand the human health implications of the rapid disruption and transformation of our planet’s natural resources that we now witness. As an example, elevated atmospheric CO_2_ concentration may result in dietary deficiencies [16]. Moreover, WHO has reported that 23% of all global deaths relate to the environment and there is evidence of how disturbances in the global environment affect global health due to disruptions of the climate system, scarcity of clean water, loss of biodiversity, land degradation, deforestation, etc. Here are some examples. In hot parts of the world, such as Central America and Sri Lanka, an epidemic of CKD (Mesoamerican nephropathy) is an emerging health concern amongst poor agricultural workers [17]. Moreover, global warming reduces gut microbiota diversity [18] and a recent study of pregnant women suggests that rising temperature is associated with perturbations in maternal heat homeostasis that increase the risk of pre-eclampsia [19]. Concerns have also been raised that global warming increases the risk of tissue hypoxia [20]. Moreover, since air pollution alters the composition of the gut microbiota in mice [21], it may not only promote respiratory disease, but also other diseases that accumulate with age. Indeed, epidemiological studies have shown links between long-term exposure of fine particulate matter (PM_2.5_) and the risk of CKD [22], obesity and type-2 diabetes [23] and cardiovascular disease [24]. The relationship between scarcity of clean water and global health, especially pertinent to gastrointestinal infections, has been evident for centuries. Lack of clean water and global warming increases the risk of chronic dehydration; a condition that may increase the risk of type-2 diabetes [25]. It has been estimated that the risk of obesity will increase by 12% from 1961 to 2081 due to global warming alone [26]. Although activation of the fructose vasopressin pathway [27] may play a major part in this, global warming will also lead to a more sedentary lifestyle and increased intake of high-calorie beverages, established risk factors for burden of lifestyle diseases. Finally, as positive associations between pollutants and obesity have been documented, it has been hypothesised that exposure to plastics, organic pollutants, heavy metals and pesticides causes obesity via damage to our natural weight control mechanisms [28].

## Inspiration from Nature Provide Innovative Ways to Improve Human Health

Due to the current environmental crisis, the human race is being challenged like never before. Thus, we must urgently find new and innovative solutions for this most challenging global problem, before it becomes humanity’s nemesis. Nature is the oldest model, measure and mentor we could ask for in our search for solutions to emerging human problems. The vast diversity of about 8.7 million species is one of the most striking aspects of life on our planet. Since the emergence of wildlife about 650 MA, animals have survived (or not) based on whether their adaptations to environmental change and the previous five mass extinctions (including global warming due to greenhouse gas emissions during the Permian–Triassic mass extinction) have been appropriate or not. Species that did not evolve and adapt to a changing environment became extinct [29]. Because humans have consistently overexploited and destroyed ecosystems and animal habitats, it will take millions of years for the planet to recover from the loss of animal diversity that is expected to occur over the next 50 years [30]. This will have a catastrophic effect, as biodiversity not only includes the quantity of currently existing species, but also the sum of the unique evolutionary developments that have taken place over geological epochs, for each individual species [30]. If Homo sapiens lives up to the name we have christened ourselves (i.e., the “wise man”), we should instead of destroying and exploiting the natural environmental balance, learn from the ingenious evolutionary adaptations and imitate them (i.e., apply a biomimetic approach) to solve prevailing human issues [31]. Although innovation-based opportunities, based on solutions in nature, have proven to be successful in a wide range of areas, such as technology, chemistry and architecture, biomedical science has not yet developed its full potential in this context [32]. The opportunities in promoting human health are staggering and biomimetic studies have already identified potential applications, such as the antibiotic and sunscreen activities of red sweat from the hippopotamus [33], marine-inspired polymers for medical adhesives [34], and inspired by micro-sized mosquitoes needles, a strategy to implant small flexible microprobes into the brain [35]. Current biological approaches focus largely on research using conventional laboratory model organisms, such as mice and rats, which take place in the unnatural laboratory environment. Recent reports have communicated doubts about the efficacy of laboratory studies in mice and rats seeking to find efficient treatment strategies for human disease. It has been argued that these models are metabolically morbid [36] and that the majority of experimentation is not valid as a consequence [37]. A biomimetic approach, using evolutionary medicine as a powerful lens, could eliminate many of these problems since nature is never careless, nor cheats in its evolutionary experiments. The risk that biomimetic research leads to similar erroneous results is thus minimal [31]. Some limitations of biomimetics should be acknowledged. As most of the existing biomimetic examples is correlative, molecular genetics should be introduced to determine causation. Moreover, it is conceivable that not all physiological and anatomical elements in animals may be the result of natural selection.

## Hibernation and Seasonal Variability Hold Clues for Human Health

There are many examples where humans can learn from wild animals, which through evolution have developed ingenious solutions for protecting themselves against chronic burden of lifestyle diseases. Hibernating bears, with a reduced metabolic state, do not develop insulin resistance or type-2 diabetes, despite pronounced obesity at the end of summer and in autumn [31]. A recent comparative study has shown that, in contrast to captive bears, free-ranging bears turn on a metabolic switch that shunts choline to generate the methyl donor betaine instead of the pro-atherogenic gut microbial metabolite trimethyl monoamine oxide (TMAO) [38]. Characterisation and understanding of how to turn on and off such a beneficial metabolic switch during stressful periods could hold clues for novel treatment options in many burden of lifestyle diseases. In addition, despite months of inactivity, starvation and impaired kidney function with anuria during hibernation, bears do not suffer from osteoporosis, muscle loss or atherosclerosis when they get back into an active lifestyle again in the spring [39]. Elevated cholesterol efflux capacity and lower affinity of LDL cholesterol for arterial proteoglycans were recently found to constitute vasculoprotective properties in bear plasma lipoproteins [40]. An upregulation of the transcription factor Nrf2 [41] and a shift in metabolic profile with a slow-oxidative fibre and mitochondrial biogenesis [42] has been shown to underlie the resistance to skeletal muscle atrophy in hibernating brown bears. High cortisol levels have been reported to be the key adaptation during hibernation, which is linked to reduced activation of AMPK/PGC-1α/PPAR-α in the regulation of metabolism in skeletal muscle and adipose tissue [43]. Finally, as the gut microbiota modulates energy metabolism in bears [44], accumulating evidence suggests that the gut microbiota plays a protective role during the vulnerable hibernation period. In contrast to the superior mechanisms that have developed during evolution to protect hibernating bears from the dramatic physiological transitions that occur between periods of high and low metabolic activity, other seasonal mammals, such as lemurs, are at higher risk, when the number of seasonal transitions increased in frequency [45]. Such studies may be of relevance for climate-induced seasonal shift and increase reasons for seasonal variation in mortality observed in burden of lifestyle diseases, such as CKD [46] and stroke [47].

## Water Conservation Systems Have Evolved over the past 350 Million Years

The link between fat accumulation and water balance in those species that suffer from water shortages for long periods, such as camels and blue whales, gives us clues about metabolic survival mechanisms that have been put out of play in a modern society with a sedentary lifestyle and overconsumption of high-calorie foods [48]. Ingenious solutions that evolved to withstand extreme temperatures in the animal kingdom may provide hints for the treatment of human diseases in an era of global warming. One of the best examples of an ingenuous solution comes from a certain beetle species in the Namib Desert that have evolved a system to collect water from the fog on their backs by way of wettability patterns [49]. The Arabian camel has developed an amazing capacity to cope with extreme heat stress and drought without any physiological impairment. Thus, camels could give us valuable indications on how to cope with global warming during the Anthropocene. Data suggest that heat shock proteins play a critical role in their tolerance to heat [50] while camel milk activates Nrf2 [51]. At the other extreme, Alaskan wood frogs can survive seasonal exposure to sub-zero temperatures and have developed amazing mechanisms to withstand interrupted blood flow and oxidative stress due to anoxia and dehydration. It was recently reported that wood frogs adapt to low-temperature stress via activation of Nrf2 and increased antioxidant defences [52]. In several recent large double-blind randomised trials, it has been demonstrated that substantial loss of glucose and sodium due to inhibition of sodium-glucose co-transporter 2 (SGLT2) has a major beneficial impact on the progression of kidney disease, major adverse cardiovascular events and hospitalisation due to heart failure and cardiovascular mortality [53]. Patients on SGLT2 inhibitor therapy adjust to the reduction in energy availability and conserve water. Estivation is an evolutionary process by which various animals, such as snails, crocodiles, hedgehogs, tortoises and lungfish, adapt to a state of dormancy during hot periods of the year to conserve energy and protect organs. As recently reviewed [54], SGLT2-inhibitors induce estivation-like metabolic patterns, which are likely to contribute to the observed improvements in cardiac and renal function. Thus, nature has already figured out a way for organ protection in estivating mammals, a metabolic switch that humans have learned to master in the 21^st^ century.

## Cancer Protective Solutions in Nature

Other wonders of nature that inspire solutions for human diseases include the amazing protection from cancer that has developed in elephants [55]. Analysis of their genome has indicated that they have increased cellular apoptotic responses to DNA damage, potentially explained by the multiple copies of the p53 gene [55], which triggers protein cell death when irreparable DNA damage is detected, which otherwise could make a cell cancerous. Humans with a mutation in *TP53* develop Li–Fraumeni syndrome and have a nearly 100% lifetime risk for developing cancer [56]. Elephants also have 11 extra copies of the leukemia inhibitory factor (*LIF*) gene, which induces apoptosis in the absence of DNA damage, or activation by the p53 gene [57]. Other mammals, such as naked mole rats, whales, grey squirrels, bats, cows and horses [58] can provide additional clues for the human fight against cancer. As an example, the capybara, a gigantic rodent native to South America, has developed an expanded family of immune-related genes that involves T cell-mediated tumour suppression, and which enhances their immune surveillance against cancer [59]. As the insulin signalling pathway allows capybara to grow large relative to other rodents, it is possible that their immune system has evolved differently compared to other rodents to compensate for the increased risk of cancer that comes with increased body size [59]. In each of these species, evolution has taken a different path to decrease the risk of cancer, which could lead to novel complementary mechanisms for cancer resistance for the development of new human cancer therapies. This is particularly pertinent, as there is a clear link between an increase in environmental pollutants and cancer. Mammals with an exceptionally high risk of cancer, such as Tasmanian devils, ferrets and dogs, may also provide insights that benefit human health. Tasmanian devils provide a “natural” disease model for exploring immune evasion mechanisms in transmissible cancer [60]. Since this cancer epidemic started in 1996, the Tasmanian devil has developed two independent lineages of allogeneic clonal transmissible facial tumour disease that has killed about 80% of the local population [61]. As the disease recently shifted from an epidemic to an endemic phase, this implies that the Tasmanian devil has developed resistance against cancer. This was recently shown to be mediated via activation of RASL11A (code for small GTPases) [62] and the major histocompatibility complex (MHC-I) that was identified as a target for anti-tumour and allogeneic immunity [63]. Moreover, as inhibition of cholesterol synthesis by atorvastatin shuts down Devil facial tumour disease energy metabolism and prevents tumour growth [64], this has provided understanding that could benefit the treatment of human cancer. Thus, elucidating the underlying mechanism of this transmissible tumour may give insights for cancer treatment. Additionally, as the phylodynamic analytical framework used to map the epidemiological dynamics of the Tasmanian devil can be applied to any pathogen, it can also guide intervention strategies in future pandemics [65].

## Organ Growth and Protection against Heart Failure in the Animal Kingdom

Nature is replete with other examples of elegant solutions for protection from burden of lifestyle diseases that could serve as biomimetic inspiration for improving human health [31]. Vertebrates have a vast array of epithelial appendages including teeth, feathers, scales, spines and hair. Over 450 million years of evolution sharks, salamanders and lizards have developed unique mechanisms for appendage regeneration of amputated or injured tissues. As explorations of this process point to customary mechanisms, such as Wnt/β-catenin and fibroblast growth factor (FGF) signalling for the restoration of a functional appendage, these animals provide a useful guide for effective regenerative strategies in man [66]. The reason(s) why larger mammals, during their evolution, lost the capacity for appendage regeneration remains elusive and requires further study. Another elegant solution needing mention is the extreme organ growth, such as the 40% cardiac hypertrophy (with increased cardiac output for about 48–72 hrs) after Burmese pythons ingest large meals, such as a goat [67]. As injection of a combination of fatty acids found in python plasma after large meals promotes physiological mammalian cardiomyocyte hypertrophy [68], targeted fatty acid supplementation may be a novel strategy to modulate cardiac gene expression and function in CHF. As a consistent enrichment of the Nrf2-mediated oxidative stress responses was recently demonstrated in the Burmese python [69], this ubiquitous cytoprotective pathway may also be important in mediating cellular stress during extreme regenerative growth.

The superior protective mechanisms against the effects of high blood pressure (twice as high as in other mammals) that have developed in the giraffe may serve as an inspiration for the development of strategies to protect humans from hypertensive-related kidney disease and CHF with preserved ejection fraction (HFpEF) [70]. One major difference in human hypertensive-induced left ventricular hypertrophy (LVH) and developmentally induced thick left ventricular wall in the giraffe is reduced cardiac fibrosis in the giraffe myocardia [71]. A recent study, which generated a high-quality giraffe genome, identified the giraffe *FGFRL1* gene as an outlier compared to other ruminants [72]. When the giraffe mutation was inserted into the *FGFRL1* gene in mice, significantly less renal and heart fibrosis was observed during hypertension [72]. Thus, as the giraffe *FGFRL1* gene counteracts the detrimental effects of hypertension, it may hold a clue for treatments to protect humans from the adverse effects of hypertension.

Early vascular aging, which is highly prevalent in patients with burden of lifestyle diseases, contributes to premature cardiovascular disease. A recent study using the transparent extracorporeal vascular network of the colonial ascidian star tunicate (*Botryllus schlosseri*) show that age-related changes, such as vessel narrowing, reduced blood flow and less responsiveness to stimuli, resemble changes that occur in aging mammalian vessels [73]. As newly regenerated vascular cells of this invertebrate maintain an aged phenotype, this suggests that heritable epigenetic vascular changes promote aging [73]. Thus, the global nature and progression of aging in star tunicate make it a new and robust model of vascular progeria studies.

## The World’s Most Unappreciated Animals may Help us Combat Antibiotic Resistance

Insects – which comprise about 85% of animal biodiversity and about 55% of total planetary biodiversity – can rapidly clear microbial infections by producing a variety of immune-induced cytokine-like molecules including antibacterial, antifungal peptides/polypeptides, which even may have anticancer activities. Indeed, antimicrobial peptides from insects have been reported to counteract antibiotic resistance [74] and prevent skin cancer [75] (as it can be formulated as ointments and creams). As antimicrobial peptides are potent at low concentrations and have high specificity with low toxicity towards normal cells, their biological properties could be utilised as novel biopharmaceuticals for both prophylactic and therapeutic applications [76]. Antibiotic resistance is a major threat to global health, which has been accelerated by the misuse of antibiotics, both in animals and humans. As gut bacteria of animals living in polluted environments, such as cockroaches, could be a potential source of anti-bacterials [77], therapeutic anti-bacterials for potential human use may already exist in some of the world’s most unappreciated animal species.

## Metabolic Magicians in Nature

The kidneys of Weddel seals are protected from hypoxia despite severe renal vasoconstriction upon extended periods of deep-see diving [78]. Although the exact mechanism(s) enabling this are unknown, it could occur through the upregulation of antioxidants. Indeed, fasting seals display increased expression of Nrf2 [79], antioxidant enzymes [80] and glutathione levels [81]. In keeping with a thesis of such a protective role for antioxidants, increased expression of Nrf2 has been reported to prevent progression of tubular damage after renal ischemic injury in mice [82]. Other amazing features that can inspire researchers to find new solutions to lifestyle diseases, such as diabetes and obesity, include the resistance to diabetic complications in hummingbirds. These small flying powerhouses possess the highest mass-specific metabolic rates known amongst vertebrates and increase their body fat >40% before migration [83]. As they almost exclusively feast on nectar sugar, they face extreme challenges to meet the high metabolic fuel requirements. Although this leads to blood glucose levels up to 42 mmol/L [84], the poor metabolic control does not seem to cause the neurological, renal and microvascular pathologies that would be found in diabetic humans [85]. The anti-obesity effects of a glucagon-like receptor agonist from the saliva of the Gila monster should also be mentioned [86].

Alcoholism is a major global health problem that can induce hepatic steatosis with liver dysfunction and oxidative stress. Nrf2 may play an important protective role against acute alcohol-induced hepatic and pancreatic damage and Nrf2-KO mice have a defective hepatic acetaldehyde (the major toxic metabolite of alcohol) metabolism [87]. Tree shrews – a small mammal native to the tropical forests of Southeast Asia – is a close relative of primates in terms of evolution. They have some dietary traits in common with humans; one of them ingestion of alcohol on a regular basis. Due to daily intake of fermented floral nectar from the Bertram Palm (the highest alcohol concentration reported in a natural food), the pen-tailed tree shrew has high concentrations of the alcohol metabolite ethyl glucuronide, but with no signs of intoxication [88]. It has been estimated that they consume intoxicating levels of alcohol about twice per week assuming alcohol metabolic rates similar to humans. The mechanisms that have evolved to protect tree shrews from repeated alcohol intoxication may provide clues for the treatment of chronic alcoholism and a model for studying alcohol-induced fatty liver disease [89]. The p53 family proteins are an evolutionarily conserved group of transcription factors that emerged at the start of multicellular life over 1 BYA [90]. p53 plays a role in protecting organisms from genotoxic stresses, in part via enhancing the protein level of Nrf2 [91]. As p53 protective mechanisms include DNA damage induced by acetaldehyde, it is of interest that the p53 protein in tree shrews is considerably more thermostable than human p53, which may lead to superior maintenance of genomic integrity and protection of acetaldehyde-induced DNA damage [92].

## Lessons from Animals with an Increased Risk of a Disease

Obligate carnivores, such as the domestic cat, tigers and lions, have a markedly increased risk of developing CKD [93,94]. Amongst geriatric domestic cats, 35%–80% develop CKD; the most common cause of death in domestic cats >5 years of age [93]. In accordance, renal injuries were reported in 87% of tigers, leopards and lions in German zoos and safari parks [94]. Given the evolutionary law of “survival of the fittest,” it is unlikely that wild felids have evolved a predisposition to CKD. Thus, the dramatic increase in CKD might reflect climate change and/or environmental change, in which felids are particularly vulnerable [95]. In contrast to domestic and zoo felids, which are often fed high-protein diets on a daily basis, dietary acquisition of protein in the wild is intermittent and separated by days of fasting without prey. As fasting drives muscular Nrf2-related antioxidant responses [96], further studies should relate to the common occurrence of fasting in the animal kingdom to survival in extreme environments. Although several different causes may explain the increased risk of CKD in felines, veterinary observations support epidemiological studies that have shown that increased intake of red meat increases the risk of CKD [97,98]. The high protein and phosphate intake that accompanies the intake of red meat and animal-based foods are likely contributory. A recent study based on the Korean Genome and Epidemiology Study concluded that in the general population, a high-protein diet increases the risk of hyperfiltration and a rapid decline in renal function [99]. Thus, high-protein regimens including meat and other animal-based foods, such as the Atkins diet, to reduce the risk of obesity and diabetes may be bad for kidney health [100]. Another culprit in felids may be hyperphosphatemia due to increased intake of phosphate that accompanies a high intake of protein. In mammals, serum phosphate level is inversely related to lifespan, thus it has been speculated that this element promotes CKD, cardiovascular disease and shortened lifespan [101]. In the general population, high dietary intake of phosphate is associated with increased all-cause mortality [42].

## Negligible Senescence and Environmental Stress – What is the Role of Nrf2?

Studies across a range of different animal species have shown that superior mitochondrial biogenesis and resistance to inflammation and oxidative stress, again via upregulation of Nrf2, protects animals in extreme habitats [31] against multi-stress genotoxicity. Indeed, in a wide variety of species, such as bats [102,103], bears [41], seals [79], the Burmese python [69] and long-lived species, such as naked mole rats [104], and ocean quahog [105], an upregulated Nrf2 system seems to be protective in an extreme natural habitat. Current evidence suggests that a common mechanism mediating outstanding stress resistance in long-lived mammals are the maintenance of protein homeostasis, superior protection against oxidative stress and robust mitochondrial function [105]. In accordance, negligible senescence in sea urchins is accompanied by a lack of accumulation of cellular oxidative damage [106]. Studies on naked mole rats [107] and hibernating bears [44] also support a role of a favourable gut microbiome in healthy aging. A protective role for Nrf2 in the aging process is further supported by low Nrf2 expression in children with extreme premature vascular aging due to Hutchinson-Gilford Progeria syndrome [108]. Additionally, a recent study conducted in Nrf2 *k/o* mice sent into space has revealed that Nrf2 deficiency induces aging-like changes in plasma metabolites and weight gain [109]. While important lessons on protective mechanisms in aging can be learned from long-lived fish with negligible senescence, such as the Greenland shark (>400 years) and Bowhead whale (>200 years), short-lived fish, such as killifish (13 weeks) have emerged as an important natural animal model for aging research [105].

While nature has allowed the evolution of homeostatic systems to counteract metabolic extremes within the body, the Nrf2 antioxidant pathway may also play a role in resistance to allostatic environmental stress. Heat extremes are increasing in frequency around our planet and affect both human and animal well-being, especially during the hot summer period. Heat stress affects normal development and differentiation at both the cellular and the organismal levels. It induces inflammation, mitochondrial damage with oxidative stress [110] and premature senescence with cell cycle arrest [111], all features of the dysregulated aging process. Many of the observed adverse health effects caused by inhaled particular matter are also associated with mitochondrial damage, oxidative stress and inflammation [112]. As ROS reacts with cellular macromolecules, including DNA and proteins, environmental stress is likely to increase the risk of mutation and protein misfolding [113]. Inflammation, mitochondrial stress and oxidative stress are closely related to the “*diseasome of aging*” [4]; that is, it is likely that global warming, air pollution and other environmental stresses will increase the incidence of burden of lifestyle diseases. In this respect, it is interesting and promising that nutritional stimulation of Nrf2 with resveratrol [114], hydrolysed camel whey protein [51] and curcumin [115] protects against thermal stress in animals. Moreover, sulforaphane – a potent nutritional Nrf2 agonist – activates heat shock transcription factor 1-mediated heat shock response [116]. Equally interesting are studies showing that Nrf2 stimulation by Juglanin isolated from knotgrass protects against prolonged PM_2.5_ exposure-triggered liver inflammation [117] and RT-408 protects against ozone-induced asthma [118]. Finally, tyrosol from olive oil prevented the toxic effects of aluminium in rats via activation of the Nrf2-Keap1 pathway [119], which has been reported as a secret weapon against pesticide persecution in Drosophila [120].

It would be naïve to think that the examples given in this text regarding the putative merits of stimulation of the cytoprotective transcription factor Nrf2 could be the one and only solution. There will never be a “silver bullet” and it should be emphasised that overstimulation of Nrf2 may have adverse effects [121]. However, considering emerging data in the literature showing the importance of this evolutionary conserved cytoprotector for animal species living in extreme environments and studies showing that Nrf2 activation protects animals from heat stress, pollution, pesticides, etc., it is important to further study the role of this system for the benefit of planetary health. It may be one of the low-hanging fruits, where the concept of “Food as medicine” may come of age and have an impact on future pandemics and non-communicable disorders alike. After all, humans have been safely ingesting nutritional Nrf2 stimulators in plant and fermented food since ancient times.

## Loss of Biodiversity Means Loss of Possible Solutions to Improve Human Health

**Fig. 4.**
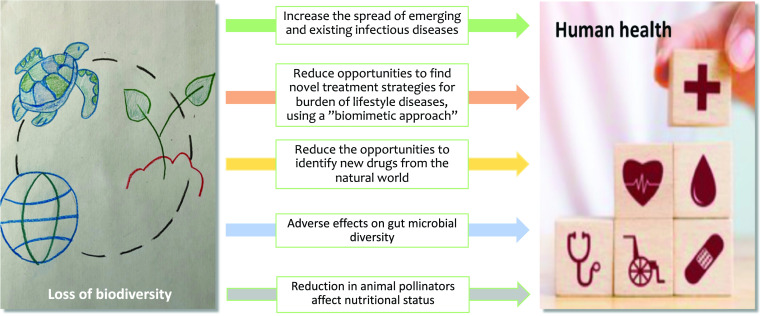
Direct or indirect mechanisms by which loss of biodiversity may affect human health.

In the “Living Planet Report 2020,” the World Wildlife Fund reported an alarming 68% decline in the animal population between 1970 and 2016 (https://livingplanet.panda.org/en-us/). This loss of biodiversity undermines the very foundations of the planet’s livelihood, health and quality of life. In a modelling analysis, Smith et al [122] reported that complete removal of animal pollinators will have a dramatic effect on global health due to micronutrient deficiencies and non-communicable diseases. The loss of biological diversity, from genetic to ecosystem level, means that we are missing out on a unique opportunity to identify biomimetic solutions to human diseases (Fig. 4). There are already multiple cases demonstrating the virtue of such solutions, a few are presented below.

One of the best examples of a successful biological biomimetic application is the development of the antihypertensive drug captopril from the poisonous Brazilian viper, whose effect on the renin–angiotensin system mimics the snake’s venom. It is likely that in the toxins from the >3000 different snakes that exist on the planet, interesting substances for drug development will be identified. As <0.01% of the planet’s snake venom has so far been identified and characterised [123], the forests of our planet are full of potentially novel drugs. The possibility of using venom from honey bees, Israeli scorpions and a marine mantle as a future treatment for cancer should also be considered [124]. About one-third of the drugs we use today have originated from nature; the development of future drugs is dependent on humankind preserving the diversity of nature. With >50,000 species of different plants in the Amazonas, it is of the utmost importance that ongoing deforestation is prevented, not only for the preservation of the environment – but also for health reasons. Clearance and disruption of Amazonian forests is one of the greatest threats to our planet’s biodiversity conservation and may increase the risk of future pandemics.

Emerging evidence suggests that in parallel with the loss of planetary biodiversity, there is a loss of human gut bacterial biodiversity. Burden of lifestyle diseases associated with the “diseasome of aging” are in general characterised by poor eating habits, dysbiosis and loss of microbial diversity [7]. A consequence of this is that bacterially derived short-chain fatty acid production will be altered, leading to epigenetic dysregulation and eventually morbid conditions, such as obesity [125]. In response to the deforestation of Amazonian forests, the majority of observed impacts on soil biodiversity, microbial biomass, richness and diversity indexes were negative [126]. Thus, loss of planetary biodiversity may drive loss of the gut bacterial biodiversity. As the plants that feed our planet need a rich and bio-diverse soil, deforestation may affect both planetary and gut biodiversity. Moreover, as plant foods are the richest source of pro-Nrf2 compounds (such as sulforaphane, physethin, curcumin and quercetin), loss of soil biodiversity and quality due to intensive monoculture and deforestation may reduce the health-promoting effects of plant food [127]. It may seem ironic now that we are beginning to receive evidence that tailor-made bioactive nutritional treatment can affect lifestyle diseases [127], this development is threatened by a disturbed external environment with global warming, greenhouse gas emission, air pollution and deforestation. The impact on burden of lifestyle diseases and gut microbial biodiversity of the global trend of migration from rural to urban areas also need attention. One recent study has shown that when sparrows migrate from rural to urban areas, this is not only associated with a loss of gut microbiota diversity, but also fewer metabolic functions in cities [128]. Another study, using functional magnetic resonance imaging, links the urban environment to vulnerability in certain brain regions and social stress [129].

What both evolution and the natural world have illustrated to mankind is that it is not advisable to stand still. Consequently, physical activity (exercise) is often a form of medicine, which can enhance physiological capabilities, both via stimulation of the immune system [130] and via upregulation of Nrf2 [131]. The sedentary lifestyle in modern man does not lend itself to the maintenance of peak physical activity. Additionally, socioeconomic factors interplay with modern lifestyles to exacerbate this situation. Indeed, neighbourhood stressors associated with socioeconomic deprivation have been linked with obesity and its sequelae, including diabetes and poor cardiovascular health, particularly amongst women. This has been attributed to a reluctance to take suitable levels of exercise when living in adverse social environments and to adopt more sedentary behaviours [132]. Biomimentics may help here by identifying more salutogenic environmental conditions that may be incorporated into urban environments.

## Has the Therapeutic Dilemma in COVID-19 Already Been Solved in Nature?

Emergence of zoonotic diseases – one of the greatest threats to biodiversity and human health – is a complex process involving not only wildlife and natural ecosystems, but also societal aspects. It has been speculated that permafrost melting due to global warming will result in the release of bacteria and virus (currently blocked in ice) may increase the risk of future epidemics [133]. The effects of the ongoing COVID-19 pandemic on human welfare, socioeconomic and political structures are, and will be, enormous. As humans destroy ecosystems through intensive land exploitation and trade in wildlife, we run the risk of being exposed to bacteria, viruses or parasites that spread between animals and humans. Climate change may have played a key role in the evolution or transmission of viruses, such as SARS-Cov-2. Indeed, changes in temperature (and other events associated with climate change) influence the reservoirs of viral infections, their transmission by insects and other vectors. It was recently reported that the Southeast Asian region is a global hotspot of climate change-driven increase in bat-borne coronaviruses [134]. It has also been suggested that there is a need to monitor Southeast Africa and South America for future pandemics, as these regions amalgamate many of the circumstances that could create a perfect storm for new hotspots [135]. For the prevention and treatment of viral pandemics, much can be learned from nature. Even before the current COVID-19 pandemic, various zoonotic diseases such as Ebola, HIV, rabies caused millions of deaths each year [136].

The therapeutic dilemma in severe COVID-19 is the combination of hyper-inflammation (cytokine storm) with a reduced interferon response [137]. In the worst-case scenario, this combination results in a pronounced vascular inflammation, endothelial dysfunction, hypercoagulability, acute respiratory distress syndrome, multi-organ failure and death [138]. Research conducted before the COVID-19 pandemic established a protective role for Nrf2 in multiple components of severe COVID-19 disease, such as respiratory distress [139], endothelial injury [140], hypercoagulability [141] and the cytokine storm [142]. COVID-19 patients with severe symptoms usually have lower interferon responses than patients with mild symptoms and SARS-CoV-2 ORF3b encodes a potent interferon antagonist [143].

Bats have over 64 MA of adaptive evolution developed features that are unique amongst mammals, such as a long lifespan relative to body size, a low rate of tumourigenesis and an exceptional ability to host viruses without presenting clinical disease [144]. As many of the planet’s ≈1200 bat species harbour viruses, such as Ebola and SARS-Cov-2, with at most mild symptoms, biomimetic inspiration for how virus infections should be handled can be obtained through studies of bats. During evolution, bats have developed an elegant solution to harbouring multiple infectious viral strains, as they control hyper-inflammation via upregulated Nrf2 expression [102], target the inflammasome pathway at multiple levels, mitigating potential immune-mediated tissue damage and disease [145], while at the same time exhibiting robust interferon-based defences. A recent study including 26 species of bats reported that DNA methylation changes are associated with innate immunity or tumourigenesis genes, reinforcing the view that bat longevity results from cancer suppression and augmented immune responses [146]. A recent clinical study has supported the biomimetic findings in bats. It has shown that robust interferon and Nrf2 systems play a significant role in protection against SARS-CoV-2; that is, the Nrf2 system was downregulated in COVID-19 patients and Nrf2 agonists (such as dimethyl fumarate) have potent interferon-independent inhibitory effects on both virus replication and hyperinflammation [147]. It is also evident that antibodies and/or mutations that inhibit the interferon response promote life-threatening cases of COVID-19 [148]. The recently reported role of the gut microbiota in interferon secretion (by *Bacteroide)* and the natural resistance against COVID-19 [149] are of major interest. Furthermore, vaccination with live vaccines (such as BCG and measles) trains our immune system and increases interferon levels, which can contribute to lower mortality [150]. Interferon production decreases with age, comorbidity [151] and stress [152]. Thus, this could partly explain why mortality in COVID-19 is related to age, comorbidity and more often affects people in socially disadvantaged areas. The mortality rate in COVID-19 is higher in areas with chronic exposure to lower air quality [153] and chronic sterile inflammation in the elderly may in itself be an important risk factor for a serious course of COVID-19 [154].

In order to survive stressful environmental conditions bats usually hibernate. Normally, viruses are harboured in bats without a high risk of spill-over to other species, but if the bat becomes stressed (e.g., becomes trapped, threatened, or undergoes an infection), the titre and spread of virus increase significantly. As an example, the intestines of virus-infected bats that were also infected with a fungus (*white-nose syndrome*) contained 60-fold more coronavirus RNA than bats with virus alone [155]. As intestinal organoids from both bats and humans can be infected by SARS-CoV-2 [156], the gastrointestinal transmission route of COVID-19 needs attention when discussing cause(s) of transmission. It is not hard to imagine that the mixing of different animal species (that normally not interact in nature) at Southeast Asian food markets could transmit viruses from bat intestines to other species [157].

The bat is the only known mammal that has developed the ability to fly. This extraordinary skill comes at an enormous expense, as 15–16 times higher metabolism is required (compared to twofold increased metabolism in birds) for them to fly. The extremely high metabolism in bats not only increases the risk of ROS generation and DNA damage, but also causes a fever of 38–41°C that may in itself reduce the viral load [73]. The high risk of DNA damage may be one reason why bats, over evolution developed increased expression of Nrf2 [102] for protection during hibernation [103] in a state of increased endoplasmic reticulum stresses [158]. The extraordinary immunology system of camelids also needs mentioning. It was recently reported that cross-reactive single-domain camelid antibodies from llama could be a potential therapeutic candidate for COVID-19 [159].

Amongst a number of potential treatments for severe COVID-19, it has been suggested that nutritional Nrf2 activation, especially when combined with transient receptor potential ankyrin 1 (TRPA1), represents a potential path out of the COVID-19 pandemic [160]. Recently, three experimental clinical cases using broccoli and glucoraphanin have provided a proof of concept, confirming the hypothesis that Nrf2-interacting nutrients are effective in COVID-19 [161]. The pro-Nrf2 protective effect of cabbage and fermented food on COVID-19 [162], is also of interest when linking nutritional habits to a “Covid-19 protective phenotype.” The emerging links between severity and immune dysfunction in COVID-19 and gut microbiota composition may provide clues for nutritional interventions that may limit the severity of the infection [163]. The benefits of nutritional Nrf2 agonists for COVID-19 (and burden of lifestyle diseases) as part of a “Food as medicine” concept [127] needs to be assessed through large studies by employing a double-blind, placebo-controlled design to stand on a robust scientific ground. It is likely that the planet will have a poor protection from future pandemics unless we protect the planet’s food systems.

## Urgent Need of a Radical Transformation in Global Food Systems

The health effects of poor dietary habits on the global burden of disease risks was recently shown in a systematic analysis including 195 countries, where it was revealed that 11 millions of deaths and 255 millions of disability-adjusted life years (DAILYs) were attributable to dietary risk factors [164]. Amongst these factors, high sodium intake, low intake of whole grains and low intake of fruits were the leading dietary factors contributing to the increased mortality rates and to the loss of DAILYs [164]. Summing up these figures, unhealthy diets (increased amount of salt, low in whole grain and fruits) is the largest global burden of disease and lead to a higher increase in morbidity and mortality than does unsafe sex, alcohol, drug and tobacco use together [165]. Thus, it is becoming painfully clear that the food systems developed in the modern world represent a “triple whammy” that not only promotes burden of lifestyle diseases, but also contributes to climate change and loss of biodiversity [166]. Processed food – the key hallmark of Western diet – promotes weight gain and drives microvascular disease and inflammation via increased intestinal barrier permeability [167]. Biomimetic studies of mammals living in extreme environments underpin the role of dietary habits, periods of fasting and the gut microbiota in survival. A carnivorous diet and processed food are detrimental to human health [168] and is associated with the “diseasome of aging” [97]. Thus, the transformation of global food systems may be the single most important factor to improve global health. Innovations in food system will also be instrumental if we should achieve the UN’s multiple Sustainable Development Goals (SDGs). Global food production is a key source of greenhouse gas emission – emitting 24% of the global total greenhouse gases (https://www.ipcc.ch/srccl/). Meeting the 1.5°C target for global warming, which is identified as critical by the UN Environment Programme, requires rapid and ambitious changes in food systems [169]. Food production is the largest water-consuming sector with the share of water withdrawn varying from 21% in Europe to 82% in Africa [165], and with a global average of 70% of water withdrawal used for irrigation. As the transformation of croplands and pastures is the largest factor causing species extinction [170], food has been considered as the single strongest lever to optimise human health and environmental sustainability [165].

A plant-based diet aligned with the concept of “Food as Medicine” [127] targeting a foodome of >25,000 substances that make up the human diet, offers an alternative path to evidence-based non-pharmacological treatments to improve both health and the environment. The effectiveness of “Food as Medicine” for the treatment of human diseases has been evident since the 17^th^ century, from when it was reported that citrus fruits prevented scurvy in sailors. Subsequent reports in the mid 20^th^ century support the use of “Food as medicine” for the treatment of a number of diseases such as pellagra, beriberi, anaemia, rickets. That the concept “Food as Medicine” has the potential to be a clinically viable route is exemplified by a study showing that the treatment with broccoli sprouts (rich in the Nrf2 agonist sulforaphane) can be as effective as metformin in difficult-to-control type-2 diabetes [171]. Moreover, urolithin A, a major microbial metabolite derived from polyphenolics of berries and pomegranate fruits, upregulates epithelial tight junction proteins through activation of Nrf2-dependent pathways to enhance gut barrier integrity [172]. An additional and important point to consider, pertinent to the modern Western diet, is the radical changes in food preservation that have occurred over history. The fermentation process (introduced during the Neolithic age to enable humans to eat “not-so-fresh” food and survive) converts components of fruits, vegetables, etc., to Nrf2 activators. As Lactobacillus bacteria, the dominant species in the fermentation process, activate Nrf2 [173], a link between healthy gut microbiota as a protection against oxidative stress and inflammation and reduced risk of disease is established. The absence of alkyl catechols from the modern diet may have serious negative consequences for Nrf2 cell defence, resulting in reduced protection against burden of lifestyle diseases [174]. However, robust clinical evidence for precise medical effects of “Food as Medicine” is still anticipated. As it was recently reported that with a combination of more plant-based human diets and reduced food waste more than two-thirds of future biodiversity losses could be avoided [175], we still have an opportunity to make a difference for the next generations.

## Next Steps – Could Biomimetics help us Improve Planetary Health?

There are several reasons why protecting the environment and the biodiversity of our planet will have profound impacts on human health. If the “wise man” wants to survive on our planet, we must gather knowledge from multiple perspectives and actively engage in studies of planetary health. The real opportunity now lies in adopting a strategy and securing funding for the whole health of the planet, where studies of human health are integrated with studies of the environment and animal health. Such studies are currently lacking, and we call for the creation of novel interdisciplinary research teams. An important obstacle is that traditional studies are funded in narrowly defined silos of current granting bodies. We call for the creation of novel granting mechanisms that transcend national and disciplinary boundaries, effectively targeting planetary health. In order to better prevent and treat the lifestyle diseases that increase with age, we can learn a lot from solutions already developed in nature. With a biomimetic approach, we should be able to learn from the ingenious solutions that have been developed in nature for the better. Unfortunately, many of our best scientific efforts today remain siloed, fragmented or rivalrous. As knowledge must be gathered from multiple perspectives, a multidisciplinary collaboration where medical doctors, natural scientists, veterinarians, climate scientists, ecologists, wildlife biologists and anthropologists meet at the intersection is required [31]. A reformed medical curriculum that includes planetary health and biomimetics would help to achieve this. If we are to adopt a more biomimetic approach in research, immediate action is required, as a rapid loss of species diversity and habitats may prevent this opportunity to learn from solutions developed in nature.
